# Relevance of structural damage in the sacroiliac joints for the functional status and spinal mobility in patients with axial spondyloarthritis: results from the German Spondyloarthritis Inception Cohort

**DOI:** 10.1186/s13075-017-1453-3

**Published:** 2017-10-24

**Authors:** Mikhail Protopopov, Joachim Sieper, Hildrun Haibel, Joachim Listing, Martin Rudwaleit, Denis Poddubnyy

**Affiliations:** 1 Department of Gastroenterology, Infectiology and Rheumatology, Charité - Universitätsmedizin Berlin, Hindenburgdamm 30, 12203 Berlin, Germany; 2 German Rheumatism Research Centre, Charitéplatz 1, 10117 Berlin, Germany; 3 Klinikum Bielefeld Rosenhöhe, An der Rosenhöhe 27, 33647 Bielefeld, Germany

**Keywords:** Axial spondyloarthritis, Ankylosing spondylitis, Structural damage, Sacroiliitis, Physical function, Spinal mobility, BASFI, BASMI

## Abstract

**Background:**

Functional status and spinal mobility in patients with axial spondyloarthritis (axSpA) are known to be determined both by disease activity and by structural damage in the spine. The impact of structural damage in the sacroiliac joints (SIJ) on physical function and spinal mobility in axSpA has not been studied so far. The objective of the study was to analyze the impact of radiographic sacroiliitis on functional status and spinal mobility in patients with axSpA.

**Methods:**

In total, 210 patients with axSpA were included in the analysis. Radiographs of SIJ obtained at baseline and after 2 years of follow up were scored by two trained readers according to the modified New York criteria grading system (grade 0–4). The mean of two readers’ scores for each joint and a sum score for both SIJ were calculated for each patient giving a sacroiliitis sum score between 0 and 8. The Bath Ankylosing Spondylitis Functional Index (BASFI) and Bath Ankylosing Spondylitis Metrology Index (BASMI) at baseline and after 2 years were used as outcome measures.

**Results:**

Longitudinal mixed model analysis adjusted for structural damage in the spine (modified Stoke Ankylosing Spondylitis Spine Score - mSASSS), disease activity (Bath Ankylosing Spondylitis Disease Activity Index - BASDAI and C-reactive protein level) and gender, revealed an independent association of the sacroiliitis sum score with the BASFI: *b* = 0.10 (95% CI 0.01–0.19) and the BASMI: *b* = 0.12 (95% CI 0.03–0.21), respectively, indicating that change by one radiographic sacroiliitis grade in one joint is associated with BASFI/BASMI worsening by 0.10/0.12 points, respectively, independently of disease activity and structural damage in the spine.

**Conclusion:**

Structural damage in the SIJ might have an impact on functional status and spinal mobility in axSpA independently of spinal structural damage and disease activity.

**Trial registration:**

ClinicalTrials.gov, NCT01277419. Registered on 14 January 2011.

## Background

Axial spondyloarthritis (axSpA) covers both patients with non-radiographic (nr) axSpA – often the earlier stage of axSpA – and patients with radiographic axSpA, also termed ankylosing spondylitis (AS). The latter is defined by the presence of structural damage in the sacroiliac joints (SIJ) on X-rays according to the modified New York criteria for AS. It was reported previously that functional status and spinal mobility parameters in patients with axSpA are independently determined by irreversible structural damage in the spine, i.e. presence of syndesmophytes and ankylosis, and by potentially reversible disease activity [1–3]. At the same time, structural damage in the SIJ (such as erosions, joint space alteration and especially ankylosis) might also have an impact on mobility and function – a hypothesis that has never been investigated in a wider range of the axSpA spectrum including nr-axSpA and AS.

In the present work, we investigated the association between structural damage in the SIJ and functional status and spinal mobility in patients with axSpA.

## Methods

### Cohort description and patient selection

The present analysis used the data of the German Spondyloarthritis Inception Cohort (GESPIC). A detailed description of the latter has been reported previously [4, 5]. Briefly, patients included in GESPIC were required to have a definite clinical diagnosis of axSpA according to the treating rheumatologist with symptom duration of up to 5 years for the non-radiographic form and up to 10 years for the radiographic form (AS). Patients with axSpA were classified as having AS if the radiographic criterion of the modified New York Criteria (presence of radiographic changes in the SIJ of at least grade II bilaterally or at least grade III unilaterally) [6] was fulfilled, and as having nr-axSpA otherwise. Overall, 210 patients were included in the present analysis based on the availability of radiographs and clinical data at baseline and after 2 years of follow up.

### Clinical assessment

Clinical assessment was performed at baseline and every 6 months thereafter until year 2. Disease activity was assessed by the Bath Ankylosing Spondylitis Disease Activity Index (BASDAI) [7] and level of C-reactive protein (CRP). Functional status was assessed by the Bath Ankylosing Spondylitis Functional Index (BASFI) [8] and spinal mobility by the Bath Ankylosing Spondylitis Metrology Index (BASMI) with an original 2-step method of calculation [9].

### Radiographic assessment

Conventional anteroposterior pelvic radiographs and lateral and anteroposterior cervical and lumbar spine radiographs were obtained locally at baseline and after 2 years of follow up. Images were digitized if not available in digitized form, anonymized and subsequently scored by two trained readers (DP and HH) who were blinded to the time point of the investigation and to all clinical data. Grading of radiographic sacroiliitis was performed according to the established scoring system used by the modified New York criteria for AS [6]:

- Grade 0 - normal
- Grade 1 - suspicious changes
- Grade 2 - minimal abnormality, small localized areas with erosion or sclerosis, without alteration in the joint width
- Grade 3 - unequivocal abnormality, moderate or advanced sacroiliitis with one or more of: erosions, evidence of sclerosis, widening, narrowing, or partial ankylosis
- Grade 4 - severe abnormality, total ankylosis

Radiographic changes in hip joints were scored on anteroposterior pelvic radiographs by the same readers using the Bath Ankylosing Spondylitis Radiology Hip Index (BASRI-hip) [10]. Structural damage in the spine was assessed by the same blinded readers according to the modified Stoke Ankylosing Spondylitis Spine Score (mSASSS), as described in detail elsewhere [11].

### Statistical analysis

For the current analysis, the mean of the scoring results of the two readers was calculated for each sacroiliac joint. Next, the sum of the two mean sacroiliitis scores (left and right SIJ) was calculated for each patient resulting in a sacroiliitis sum score between 0 (no signs of radiographic sacroiliitis in either SIJ in the opinion of both readers) and 8 (total ankylosis in both SIJ in the opinion of both readers) at baseline and after 2 years. To visualize an association between BASFI/BASMI and the sacroiliitis sum score, combined scatter and cumulative probability plots were created.

The association between the sacroiliitis sum score and BASFI/BASMI was explored first in the linear regression analysis, which was performed with baseline values of the variables of interest. Crude unstandardized parameter estimates (ß coefficients) obtained in the univariable model were then adjusted in the multivariable model for the following factors considered as potential confounders: structural damage in the spine (mSASSS), disease activity (BASDAI and CRP), and sex. In order to address the question of the possible confounding of the association between radiographic sacroiliitis and function/spinal mobility by the damage in the hip joints, models with inclusion of the BASRI-hip as a covariate were built.

Next, linear regression analysis of the association between change in BASFI/BASMI and progression of radiographic sacroiliitis defined as (1) absolute change in the sacroiliitis sum score, (2) worsening of radiographic sacroiliitis by at least one grade in one sacroiliac joint in opinion of both readers and (3) progression from nr-axSpA to AS in the opinion of both readers was performed.

At the final step, both baseline and year-2 parameters were included in a longitudinal linear mixed model analysis that was corrected for the dependencies between the two time-point values of each individual. Crude parameter estimates were adjusted for the same potential confounders as in the linear regression analysis. The latter analysis was performed in the entire group and also separately in the non-radiographic and radiographic subgroups of the patient population in order to address the question of a possibly higher functional relevance of the more advanced structural changes in the SIJ; 95% confidence intervals (CIs) for the parameter estimates were calculated. The inter-reader agreement was assessed by means of the weighted Kappa value. Statistical analysis was performed using SAS v.9.4 (SAS Institute Inc., Cary, NC, USA).

## Results

There were no major differences in general baseline characteristics between the included patients (radiographic cohort) and the whole previously described cohort [4], except for a somewhat lower percentage of men in the nr-axSpA subgroup (33.7% in the current study vs 42.9% in the whole cohort). General characteristics of the patients at baseline are presented in Table 1.

**Table 1:** Baseline disease characteristics of 210 patients with axial spondyloarthritis

Baseline parameters	Nr-axSpA (n = 95)	AS (n = 115)	All patients (n = 210)
Age, years	38.7 ± 9.9	36.8 ± 11.0	37.3 ± 10.6
Male gender, *n* (%)	32 (33.7)	75 (65.2)	107 (51.0)
HLA-B27 positive, *n* (%)	69 (72.6)	97 (84.3)	166 (79.0)
Duration of symptoms, years	3.2 ± 2.2	5.2 ± 2.8	4.2 ± 2.7
BASDAI, points NRS (0–10)	4.2 ± 2.0	3.8 ± 2.2	3.9 ± 2.2
Peripheral arthritis, *n* (%)	16 (16.8)	15 (13.0)	31 (14.8)
BASFI, points NRS (0–10)	2.8 ± 2.2	3.0 ± 2.4	2.9 ± 2.3
BASMI, points (0–10)	1.5 ± 1.5	2.0 ± 1.7	1.8 ± 1.6
CRP, mg/l	6.5 ± 12.8	12.4 ± 16.6	9.7 ± 15.2
mSASSS, points (0–72)	2.3 ± 4.2	5.9 ± 10.3	4.2 ± 8.3
NSAIDs intake, *n* (%)	64 (67.4)	76 (66.1)	140 (66.7)
Systemic steroids intake, *n* (%)	6 (6.3)	6 (5.2)	12 (5.7)
DMARDs intake, *n* (%)	26 (27.4)	35 (30.4)	61 (29.0)
TNFα blocker intake, *n* (%)	1 (1.1)	4 (3.5)	5 (2.4)
Current smoking, *n* (%)	24 (25.3)	39 (33.9)	63 (30.0)

Continuous variables are presented as mean ± standard deviation

*AS* ankylosing spondylitis, *BASDAI* Bath Ankylosing Spondylitis Disease Activity Index, *BASFI* Bath Ankylosing Spondylitis Functional Index, *BASMI* Bath Ankylosing Spondylitis Metrology Index, *CRP* C-reactive protein, *DMARDs* disease-modifying anti-rheumatic drugs, *mSASSS* modified Stoke Ankylosing Spondylitis Spine Score, *nr-axSpA* non-radiographic axial spondyloarthritis, *NRS* numeric rating scale, *NSAIDs* non-steroidal anti-inflammatory drugs, *TNFα* tumour necrosis factor alpha

**Fig. 1:**
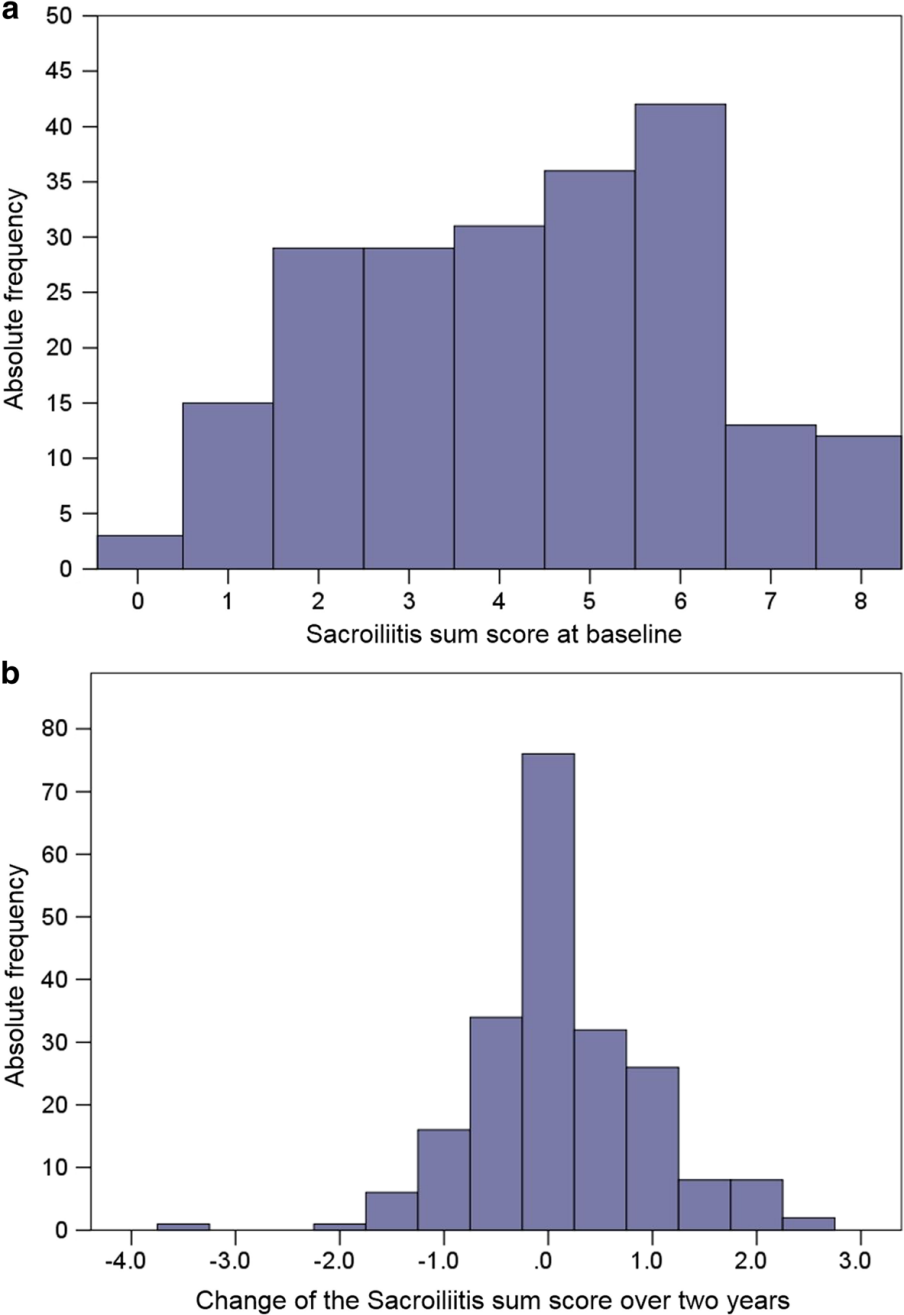
Distribution of the sacroiliitis sum score at baseline (**a**) and change in the sacroiliitis sum score over 2 years (**b**) in 210 patients with axial spondyloarthritis

Detailed results of the assessment of the SIJ on pelvic radiographs including inter-reader variability and change over time have been reported previously [5, 12]. Briefly, based on the baseline assessment of the SIJ, 115 out of 210 patients were classified as having AS, and 95 as having nr-axSpA. The sacroiliitis sum score distribution at baseline and change in the sacroiliitis sum score after 2 years of follow up are presented in Fig. 1. The mean sacroiliitis sum score at baseline was 4.3 ± 2.0 and the mean change in the sacroiliitis sum score over 2 years was 0.13 ± 0.84. In the entire group of 210 patients, a total of 26 patients (12.4%) had progression of radiographic sacroiliitis by at least one grade after 2 years in the opinion of both readers (16.8% of patients with nr-axSpA and 8.7% of patients with AS). Improvement in radiographic sacroiliitis by at least one grade in the opinion of both readers was observed in 11 patients (5.2%) in the entire group (6.3% and 4.4% of patients with nr-axSpA and AS, respectively). Progression from nr-axSpA to AS after 2 years was observed in the opinion of both readers in 11 out of 95 patients (11.6%), and regression from AS to nr-axSpA was observed in 3 out of 115 patients (2.6%).

**Fig. 2:**
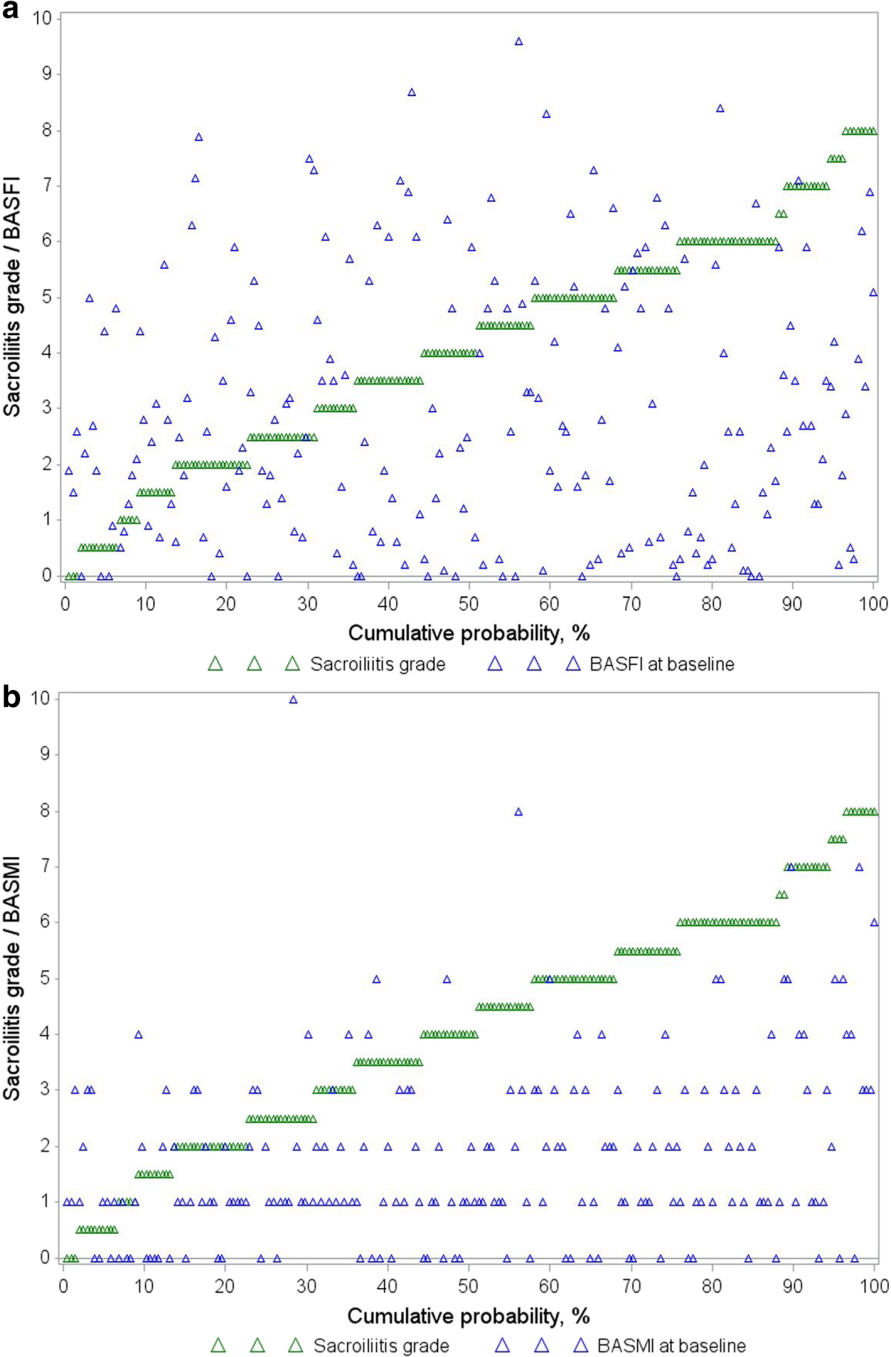
Combined cumulative probability and scatter plots depicting an association between the sacroiliitis sum score and the Bath Ankylosing Spondylitis Functional Index (BASFI) (**a**) or the Bath Ankylosing Spondylitis Metrology Index (BASMI) (**b**) at baseline in 210 patients with axial spondyloarthritis

There was moderate agreement between the two readers on the fulfilment of the modified New York criteria (weighted Kappa value 0.59 at baseline, and 0.67 and after 2 years of follow up) and on the grade of radiographic sacroiliitis (weighted Kappa values between 0.51 and 0.59 for the left and right SIJ at baseline and year 2) [5]. The combined cumulative probability and scatter plots on Fig. 2 depict the crude relationship between the sacroiliitis sum score and BASFI (Fig. 2a) or BASMI (Fig. 2b).

In the univariable linear regression models, the crude parameter estimates (ß) for the association between sacroiliitis sum score and BASFI/BASMI at baseline were 0.09 (95% CI -0.07 to 0.25) and 0.22 (95% CI 0.12 to 0.33), respectively. After adjustment for the mSASSS, disease activity parameters (BASDAI and CRP), and sex, ß changed to 0.10 (95% CI -0.01 to 0.21) and 0.12 (95% CI 0.01 to 0.22), respectively. These parameter estimates indicate that difference in one sacroiliitis grade is responsible for the difference in 0.10/0.12 points on the BASFI/BASMI, respectively. Adding the BASRI-hip score as an additional potential confounder in the models did not substantially affect the parameter estimates for either functional outcome (ß = 0.11, 95% CI -0.01 to 0.23 and 0.13, 95% CI 0.02 to 0.24 for BASFI and BASMI, respectively), therefore, this variable was not included in further analysis.

In the linear regression analysis of the association between BASFI/BASMI change scores over 2 years with the absolute change in the sacroiliitis sum score, ß was 0.03 (95% CI -0.19 to 0.25) and 0.25 (95% CI 0.03 to 0.47), respectively, adjusted for the mSASSS change score, BASDAI change score, time-averaged CRP over two years, and sex.

Worsening of radiographic sacroiliitis by at least one grade in the opinion of both readers was assessed in a similar analysis, with ß of -0.13 (95% CI -0.69 to 0.42) and 0.64 (95% CI 0.09 to 1.19), for BASFI and BASMI, respectively. Progression from nr-axSpA to AS after 2 years was assessed in 95 patients classified as having nr-axSpA at baseline, with ß of 0.79 (95% CI -0.06 to 1.65) and 0.49 (95% CI -0.04 to 1.35), for BASFI and BASMI, respectively.

In the longitudinal mixed model analysis (Table 2) that included values obtained at both time points (baseline and year 2) with correction for dependencies between two values related to the same patient, the sacroiliitis sum score demonstrated stronger association with the BASMI than with the BASFI, with ß = 0.20 (95% CI 0.11 to 0.30) and 0.09 (95% CI -0.05 to 0.22), respectively, in the univariable analysis. However, after adjustment for the mSASSS, disease activity parameters (BASDAI and CRP) and gender, the strength of association became very similar for both outcomes: ß = 0.10 (95% CI 0.01 to 0.19) for BASFI and ß = 0.12 (95% CI 0.03 to 0.21) for BASMI, indicating that progression by one radiographic sacroiliitis grade would result in a change of 0.10/0.12 points in the BASMI/BASFI. Therefore, progression from grade 0 bilaterally to grade 2 bilaterally or grade 4 bilaterally would result in an increase of 0.40/0.48 or 0.80/0.96 points on the BASFI/BASMI, respectively, independently of structural damage in the spine and disease activity.

**Table 2:** Association between the sum radiographic sacroiliitis score and functional status/spinal mobility in patients with axial spondyloarthritis

Parameters	Unadjusted mixed model analysis (all patients, n = 210), B (95% CI)	Adjusted mixed model analysis (all patients, n = 210), B (95% CI)	Adjusted mixed model analysis (patients with AS, n = 105), B (95% CI)	Adjusted mixed model analysis (patients with nr-axSpA, n = 95), B (95% CI)
Outcome: BASFI				
Sacroiliitis sum score (0–8)	0.09 (-0.05 to 0.22)	**0.10 (0.01 to 0.19)**	0.16 (-0.03 to 0.36)	0.09 (-0.11 to 0.30)
mSASSS, points (0–72)	-	0.05 (0.03 to 0.07)	0.04 (0.02 to 0.06)	0.08 (0.03 to 0.14)
BASDAI, points NRS (0–10)	-	0.81 (0.74 to 0.88)	0.84 (0.74 to 0.93)	0.75 (0.63 to 0.86)
CRP, mg/l	-	0.00 (-0.01 to 0.01)	0.00 (-0.01 to 0.01)	0.00 (-0.02 to 0.01)
Male sex	-	-0.02 (-0.40 to 0.37)	0.03 (-0.48 to 0.55)	-0.13 (-0.74 to 0.48)
Outcome: BASMI				
Sacroiliitis sum score (0–8)	0.20 (0.11 to 0.30)	**0.12 (0.03 to 0.21)**	0.17 (-0.02 to 0.37)	0.21 (0.01 to 0.40)
mSASSS, points (0–72)	-	0.07 (0.05 to 0.09)	0.07 (0.04 to 0.09)	0.04 (-0.01 to 0.10)
BASDAI, points NRS (0–10)	-	0.22 (0.15 to 0.29)	0.23 (0.14 to 0.32)	0.18 (0.07 to 0.30)
CRP, mg/l	-	0.01 (0.00 to 0.02)	0.01 (0.00 to 0.03)	0.00 (-0.03 to 0.01)
Male sex	-	0.00 (-0.39 to 0.39)	0.32 (-0.21 to 0.85)	-0.35 (-0.92 to 0.22)

*mSASSS* modified Stoke Ankylosing Spondylitis Spine Score, *AS* Ankylosing Spondylitis, *nr-axSpA* non-radiographic axial spondyloarthritis, *BASFI* Bath Ankylosing Spondylitis Functional Index, *CRP* C-reactive protein, *BASDAI* Bath Ankylosing Spondylitis Disease Activity Index, *NRS* numerical rating scale, *BASMI* Bath Ankylosing Spondylitis Metrology Index

Main results are marked in bold

Similar analysis performed in the AS and nr-axSpA subgroups resulted in similar results, though the precision of the parameter estimation decreased due to the smaller group size (Table 2)*.*

## Discussion

In this work, we showed that structural damage in the SIJ might have an impact, although small, on spinal mobility and physical function in patients with axSpA, independently of other parameters, such as disease activity and structural damage in the spine. To our knowledge, this is the first study that addresses this question in a population of patients with axSpA including non-radiographic and radiographic axSpA (i.e., the full range of radiographic sacroiliitis) and using standardized measures of spinal mobility and functional status assessment – BASMI and BASFI.

Only a few studies have investigated the potential impact of radiographic sacroiliitis on mobility and functional status in patients with axSpA or AS. In a study by Kennedy et al., an association between BASMI score and total radiological score (incorporating the radiographic grade of sacroiliitis) in patients with AS was shown, although the association between radiographic sacroiliitis and spinal mobility measures, regardless of spinal damage, was not investigated [13]. A study by Viitanen et al. showed correlation between radiographic changes in the lumbar spine and in the SIJ and spinal mobility measures, but again the effect of radiographic sacroiliitis alone was not estimated [14]. Taylor et al. also observed correlation between spinal flexion and the SIJ ankylosis score assessed on computed tomography that was, however, not adjusted for any confounders [15]. In a study in reactive arthritis (Reiter’s syndrome) radiographic sacroiliitis was found to be mostly asymptomatic and functionally irrelevant, but no standardized methods of spinal mobility and functional status assessment were used [16]. A more recent population-based study revealed no difference in the patient-reported outcome between subjects with and without radiographic sacroiliitis, but again, no standardized methods of spinal mobility and functional status assessment were used, and the number of cases with sacroiliitis was small (n = 14) [17].

Several studies investigated the influence of artificial SIJ ankylosis on spinal mobility. For example, no difference was identified between patients who underwent unilateral and bilateral transsacral-transiliac screw fixation in terms of functional status, indicating that artificial ankylosis of the second SIJ does not reduce the functional parameters (although the outcome was not an objective measurement, but rather a self-reported outcome) [18]. Comparison of quality of life and performance in patients who underwent unilateral SIJ fusion showed results comparable with the average health status of the US population, indicating no significant influence of unilateral SIJ fusion on functional performance [19]. Importantly, SIJ fusion is normally performed in symptomatic patients with mechanical problems in the SIJ and even an improvement in function and spinal mobility could be expected due to pain reduction as an outcome of the intervention.

In our analysis, both cross-sectional and longitudinal models showed a rather small but significant and independent association between radiographic sacroiliitis and spinal mobility/function in patients with axSpA (though the precision of the effect estimation was better in the longitudinal model), indicating that progression from normal (without structural damage visible on radiographs) SIJ to complete bilateral ankylosis would result in a worsening of the BASFI/BASMI by about 1 point (on a 0 − 10 scale), independently of disease activity and structural damage in the spine. For comparison, worsening of the mSASSS score by 1 point (on a scale between 0 and 72) has been reported to result in worsening of the BASFI by 0.06 points, independently of the BASDAI [2].

We hypothesize several explanations for our findings. The first possible explanation would be that the observed independent association between structural damage in the SIJ and spinal mobility/functional status is real. In this case, we have to postulate that the SIJs are indeed relevant for spinal mobility, and structural damage in these joints results in lower function. If so, stopping structural damage in the SIJ might become an additional treatment target in axSpA aiming at preservation of the spinal mobility/functional status in axSpA in a long-term perspective. Nevertheless, a benefit/risk assessment of intensified treatment would be necessary in this case.

An alternative explanation might be related to the fact that the mSASSS score takes into account only structural damage involving the anterior corners of the cervical and lumbar vertebrae. Although mSASSS is considered to be a proxy for the structural damage in the entire spinal column, the fact that assessment of damage to the spinal structures is not included in the mSASSS (i.e. the posterior structures, thoracic spine) might be responsible for the residual confounding causing a significant association between radiographic sacroiliitis and the BASFI/BASMI after adjustment for the mSASSS. We also cannot exclude residual confounding related to other factors, which were not captured and therefore not included in the analysis (e.g. effects of inflammation in the spine as detected by magnetic resonance imaging, which was not performed in GESPIC in the first 2 years).

The clinical significance of our findings is ambiguous. On the one hand, the results expressed in absolute numbers (the change in one sacroiliitis grade in one of the joints is associated with a change of 0.10/0.12 points in the BASFI/BASMI, respectively) seem quite modest, especially considering that the rate of radiographic progression is slow [5, 20–22]. On the other hand, this association is independent from the other factors, somewhat higher than expected and, if true, alongside with other factors it might be a significant contribution to the deterioration of physical function in the long run.

## Conclusion

Data from GESPIC indicate that structural damage in the SIJ might have an impact on functional status and spinal mobility independently of structural damage in the spine and disease activity in patients with axSpA – an association that has been mostly neglected previously.

## Abbreviations

AS: Ankylosing spondylitis
axSpA: Axial spondyloarthritis
BASDAI: Bath Ankylosing Spondylitis Disease Activity Index
BASFI: Bath Ankylosing Spondylitis Functional Index
BASMI: Bath Ankylosing Spondylitis Metrology Index
BASRI-hip: Bath Ankylosing Spondylitis Radiology Hip Index
CIs: 95% Confidence intervals
CRP: C-reactive protein
GESPIC: German Spondyloarthritis inception cohort
mSASSS: Modified Stoke Ankylosing Spondylitis Spine Score
nr-axSpA: Non-radiographic axial spondyloarthritis
SIJ: sacroiliac joints
